# Supplementary material for: Mechanisms governing the pioneering and redistribution capabilities of the non-classical pioneer PU.1
Source: Nat Commun. 2020 Jan 21;11:402. doi: 10.1038/s41467-019-13960-2 (PMC6972792; doi:10.1038/s41467-019-13960-2)
Supplement: Supplementary file 7 — Source data [file 41467_2019_13960_MOESM7_ESM.zip › Source_Data/Figure6/Figure6B_MotifScanOutput/ETS1_PU.1/homerResults/motif5.similar.html]

motif5

## Information for motif5

A
T
C
G
A
C
G
T
C
G
A
T
G
A
T
C
A
G
T
C
A
G
T
C
A
G
T
C
G
C
A
T
A
G
C
T
G
A
C
T
A
G
C
T
  
Reverse Opposite:  

T
C
G
A
C
T
G
A
T
C
G
A
C
G
T
A
T
C
A
G
T
C
A
G
C
T
A
G
C
T
A
G
G
C
T
A
G
T
C
A
T
A
G
C
  

|  |  |
| --- | --- |
| p-value: | 1e-619 |
| log p-value: | -1.428e+03 |
| Information Content per bp: | 1.728 |
| Number of Target Sequences with motif | 4155.0 |
| Percentage of Target Sequences with motif | 26.25% |
| Number of Background Sequences with motif | 2485.8 |
| Percentage of Background Sequences with motif | 7.75% |
| Average Position of motif in Targets | 100.2 +/- 53.1bp |
| Average Position of motif in Background | 100.5 +/- 75.9bp |
| Strand Bias (log2 ratio + to - strand density) | 0.1 |
| Multiplicity (# of sites on avg that occur together) | 1.14 |
| Motif File: | file (matrix) reverse opposite |

### Similar de novo motifs found

|  |  |  |  |  |  |  |  |
| --- | --- | --- | --- | --- | --- | --- | --- |
| Rank | Match Score | Redundant Motif | P-value | log P-value | % of Targets | % of Background | Motif file |
| 1 | 0.879 | C A T G A T C G C G A T A C G T T A G C G A C T A C G T A G T C G C A T A C G T A G C T A G C T A C G T | 1e-414 | -955.047382 | 22.14% | 7.69% | motif file (matrix) |
| 2 | 0.860 | C T G A C T G A C T G A C T G A C T G A C T G A A T C G C T A G C T A G C G T A C G T A T A C G A G C T | 1e-281 | -647.552525 | 16.21% | 5.78% | motif file (matrix) |
| 3 | 0.789 | T C G A C T G A C T G A C G T A C G T A C T A G A T G C C G T A A C T G G T C A C G T A T A C G G A C T C G T A | 1e-101 | -232.676630 | 5.24% | 1.65% | motif file (matrix) |
| 4 | 0.809 | C G T A C G T A C G T A A C T G C T A G A C T G C G T A C G T A | 1e-56 | -130.871275 | 11.40% | 6.99% | motif file (matrix) |
| 5 | 0.790 | G C A T G A C T T G A C G A T C G A T C G T A C C G A T G C A T A C G T T A G C G C A T T A G C | 1e-27 | -64.132398 | 0.94% | 0.20% | motif file (matrix) |
